# Lifespan Perspectives on Gynecologic and Obstetric Pathologies: Reflections and Frontiers

**DOI:** 10.3390/jcm14196927

**Published:** 2025-09-30

**Authors:** Panagiotis Christopoulos, Ermioni Tsarna

**Affiliations:** Second Department of Obstetrics and Gynecology, School of Medicine, ‘Aretaieion’ Hospital, National and Kapodistrian University of Athens, 11528 Athens, Greece

## 1. Prenatal Risk Stratification and Obstetric Screening

Clinical obstetrics and gynecology increasingly rely on an interdisciplinary synthesis of a wide range of scientific disciplines, including molecular biology, genomics, embryology, environmental health sciences, biochemistry, pathophysiology, endocrinology, and behavioral and health psychology. Advances in all these fields have continually contributed to the base of evidence that underlies clinical practice. Nonetheless, the repeated failure to successfully implement documented preventive measures, such as limiting prenatal exposure to well-documented teratogens and stressors, continues to result in avoidable adverse outcomes. Therefore, clinicians have a dual responsibility: to incorporate new scientific knowledge while reinforcing pre-existing knowledge with the aim to prevent long-term pathologies, interrupt negative intergenerational cycles, expand therapeutic options, support individualized reproductive decision making, address psychological complaints, and optimize healthy aging through proactive management of reproductive risk factors. As we conclude the second edition of the Special Issue in the *Journal of Clinical Medicine*, Gynecologic and Obstetric Pathologies from Birth to Menopause, this editorial examines contemporary reflections and delineates future directions in gynecologic and obstetric disease across the female lifespan.

In modern obstetric practice, prenatal diagnostics and accurate risk stratification are essential to guide clinical decision making and facilitate secondary prevention strategies. Given the potential complications associated with invasive prenatal procedures such as amniocentesis, there has been growing interest in the development and implementation of non-invasive approaches (Contribution 3). Twin pregnancies, however, present unique complexities in non-invasive prenatal screening due to the possibility of discordant outcomes between co-twins. A systematic review evaluating the predictive utility of pregnancy-associated plasma protein-A (PAPP-A) in twin gestations demonstrated associations between reduced PAPP-A levels and an increased risk of preterm birth and fetal growth restriction, though not with hypertensive disorders of pregnancy (Contribution 11). The prediction of hypertensive disorders—particularly pre-eclampsia—has become an area of intensified research focus in recent years. Among existing tools, the Fetal Medicine Foundation (FMF) competing-risks model for preterm pre-eclampsia, which incorporates maternal characteristics, blood pressure measurements, and biochemical markers, remains the most thoroughly validated and high-performing prediction model to date [1]. Nonetheless, the prediction of late-onset pre-eclampsia continues to be challenging, highlighting the need for a deeper understanding of its underlying pathophysiology and the discovery of novel predictive biomarkers. In this context, differential adaptations in the Renin–Angiotensin–Aldosterone System (RAAS) between normotensive and pre-eclampsia-affected pregnancies have emerged as a promising area of investigation (Contribution 7).

## 2. Modifiable Risk Factors and Lifestyle Influences

Exposures during pregnancy, such as maternal smoking, continue to exert significant adverse effects. Dose-dependent associations have been documented between smoking and outcomes including stillbirth, congenital anomalies, and intrauterine growth restriction [2,3,4]. Long-term sequelae—such as asthma, obesity, and impaired cognition—are also linked to in utero smoke exposure [5]. Furthermore, maternal smoking during pregnancy frequently coexists with other established risk factors for poor perinatal outcomes, including maternal obesity and multiparity (Contribution 3). The aforementioned risks appear to be exacerbated in twin pregnancies, where nicotine-induced vasoconstriction and carbon monoxide exposure contribute to heightened fetal hypoxia, owing to the increased physiological demands of multiple gestations (Contribution 8). Nevertheless, pharmacological interventions for smoking cessation during pregnancy remain insufficiently investigated. Limited safety data and inconclusive evidence regarding their impact on birth outcomes continue to pose barriers to broader clinical adoption [6].

## 3. Modes of Delivery: Clinical and Systemic Determinants

The mode of delivery has been acknowledged as a determinant of long-term health outcomes for both mothers and their offspring. The World Health Organization (WHO) has highlighted significant global disparities in access to cesarean section (CS), with certain regions facing limited availability while others exhibit excessive utilization—sometimes surpassing vaginal birth rates [7]. Both underuse and overuse of CS are associated with preventable maternal and neonatal morbidity and mortality [7,8]. In this Special Issue, factors contributing to elevated CS rates were examined in Greece, where the CS rate reached 62.15% in 2023 (Contribution 1). Medicolegal pressures, a high prevalence of high-risk and “precious” pregnancies, insufficient training in instrumental vaginal delivery techniques, and maternal preferences emerged as key drivers. These findings highlight critical areas for targeted interventions aimed at optimizing delivery practices and reducing unnecessary CS rates.

## 4. Infectious Disease and Disparities

Global inequities in maternal and neonatal health are evident across a range of clinical outcomes and public health indicators. Among these, the burden of maternal infections, sepsis, and related adverse outcomes, including maternal and neonatal mortality, remains substantial. These conditions disproportionately affect regions with a lower sociodemographic index (SDI), a disparity largely attributed to inadequacies in healthcare infrastructure and limited access to routine antenatal care [9]. As incidence rates continue to decline in high-SDI settings, healthcare professionals in these regions may encounter diagnostic challenges, particularly in recognizing atypical infections that present with non-specific clinical manifestations (Contribution 9). A notable example is the first documented case of visceral leishmaniasis in a twin pregnancy, in which prompt diagnosis and treatment with liposomal amphotericin-B led to favorable perinatal outcomes, with no evidence of vertical transmission or recurrence at one-year follow-up (Contribution 9).

## 5. Contraceptive Safety and Complications

Intrauterine devices (IUDs) represent one of the most effective and reliable methods of reversible contraception, playing a significant role in reducing the incidence of unintended pregnancies. However, on rare occasions, IUD migration can occur, frequently involving the abdominal cavity and urinary tract, situations in which prompt removal of the displaced device is recommended [10]. Detection of migrated IUDs in older patients is uncommon and may present greater challenges during removal due to the formation of adhesions (Contribution 12). Regular monitoring of IUD position, coupled with patient self-inspection of IUD strings intravaginally, may facilitate earlier identification of asymptomatic or oligosymptomatic uterine perforation and device migration.

## 6. Benign Disease and Onco-Risk Spectrum

Endometriosis is a benign gynecological condition commonly presenting with dysmenorrhea, dyspareunia, and infertility among women of reproductive age. Nevertheless, advancing age in patients with endometriosis is associated with an increased risk of ovarian cancer, particularly the endometrioid and clear cell histological subtypes [11]. Endometriosis-associated ovarian cancer (EAOC) patients often present at younger ages, earlier stages, and with lower-grade tumors, leading to improved overall survival despite similar progression-free survival compared to non-EAOC cases [11]. A recent systematic review identified genetic and epigenetic modifications in EAOC, implicating oxidative stress and chronic inflammation as key mechanisms in its pathogenesis (Contribution 10). Recognized risk factors for malignant transformation include the presence of large ovarian endometriomas, post-menopausal status, and prolonged estrogen exposure (Contribution 10). Encouragingly, advancements in diagnostic modalities such as magnetic resonance relaxometry, alongside machine learning-based risk prediction models, have demonstrated potential for early EAOC detection, thereby enhancing prospects for survival and quality of life (Contribution 10).

## 7. Patient-Centered Outcomes as Core Metrics

Health-related quality of life (QoL) and patient-reported outcomes (PROs) have increasingly been acknowledged as essential components in enhancing the interpretability of clinical findings, facilitating patient-centered decision making and care, and informing health policy formulation [12]. The use of validated, standardized tools is critical, with attention to cross-cultural and multilingual validation [12]. In this Special Issue of the *Journal of Clinical Medicine*, contributions addressing the validation of QoL-related questionnaires—specifically in relation to urinary incontinence and dysmenorrhea—have been included (Contributions 4 and 5). Beyond descriptive measurement, linking PROs to biological markers can elucidate the pathophysiology underlying patient symptoms. An illustrative study found that peri- and post-menopausal women reporting hot flashes had poorer sleep quality and elevated—but not statistically significant—levels of LINE-1 and Alu DNA methylation (Contribution 6). Notably, these methylation markers and hot flashes have separately been associated with cardiovascular risk.

In conclusion, the contributions to the Special Issue Gynecologic and Obstetric Pathologies from Birth to Menopause of the *Journal of Clinical Medicine* collectively highlight the importance of adopting a lifespan perspective in obstetric and gynecologic research—one that integrates risk stratification, modifiable exposures, the continuum from benign to malignant disease, and patient-centered outcomes. Looking forward, and in line with the multidimensional scope of modern obstetrics and gynecology, several priorities emerge for future research. These include a focus on high-prevalence obstetric and gynecologic conditions through the integration of biomarkers, advanced imaging technologies, and patient-reported outcomes (PROs). Scientific efforts should also prioritize equity-driven interventions to reduce disparities across different sociodemographic index (SDI) settings and longitudinal cohort studies that combine molecular biomarkers with PROs to elucidate causal mechanisms and support personalized prevention strategies. Additionally, the cross-cultural and multilingual validation of quality-of-life instruments tailored to gynecologic and obstetric care remains a critical goal for ensuring global relevance. By aligning scientific rigor with patient perspectives and systemic realities of healthcare systems, future research can substantially advance prevention, diagnosis, and clinical care—ultimately improving health outcomes for women across the lifespan.
